# Adapted physical activity and cardiac coherence in hematologic patients (APACCHE): study protocol for a randomized controlled trial

**DOI:** 10.1186/s13102-020-00170-3

**Published:** 2020-03-14

**Authors:** Claire Fournié, Nicolas Bouscaren, Georges Dalleau, Victorine Lenclume, Catherine Mohr, Patricia Zunic, Quentin Cabrera, Chantal Verkindt

**Affiliations:** 1 Laboratoire IRISSE EA4075, UFR des Sciences de l’Homme et de l’Environnement, Université de la Réunion, Le Tampon, La Réunion France; 2 Centre d’Investigation Clinique, Inserm CIC 1410, CHU Sud Réunion, Saint-Pierre, La Réunion France; 3 Service d’Hématologie Clinique, CHU Sud Réunion, Saint Pierre, La Réunion France

**Keywords:** Cancer, Hematologic patients, Supportive cancer care, Physical exercise, Adapted physical activity, Cardiac coherence, Heart rate variability biofeedback, Quality of life

## Abstract

**Background:**

Hematologic malignancies and their treatments are recognized for their significant long-term adverse effects on health-related quality of life. As a part of cancer treatment, physical exercise is known to improve physical functioning, but there are still questions regarding its impact on psychological and emotional functioning. Nonetheless, heart rate variability biofeedback (HRVB) is recognized for its positive effects on autonomic nervous system balance and emotional self-regulation. The Adapted Physical Activity and Cardiac Coherence in Hematologic Patients (APACCHE) protocol is a randomized, controlled superiority trial designed to evaluate the effects of HRBV training combined with an adaptive physical activity (APA) program compared to APA alone on the post-treatment quality of life of adult hematologic patients.

**Methods:**

Seventy patients aged 18–70 years, with various forms of hematological malignancies, in post-treatment remission within six months prior to beginning the study and who have been prescribed APA by a hematologist, will be randomly allocated in a 1:1 ratio to two 12-week treatment groups: HRVB + APA versus APA alone. APA sessions will consist of aerobic and resistance training for 1-h twice weekly. The HRVB training will consist of controlled breathing exercises with biofeedback of heart rate variability for 10 sessions and will include a daily home-based practice. The primary outcome will be to evaluate health-related quality of life (QLQ-C30, SF-36). The secondary outcomes will be to evaluate fatigue (MFI-20); anxiety and depression (HADS); clinical status with blood pressure, progression-free survival, overall survival, and body mass index; heart rate variability level and cardiac coherence score. All of these assessments will be evaluated initially (T1), 6 weeks after (T2), at the end of the 12 weeks (T3), and then at a 12-week post-intervention follow-up (T4).

**Discussion:**

To our knowledge, this is the first protocol to investigate the additional value of HRVB on physical exercise. In addition, there has been no study previously published about HRVB in hematologic patients. We hypothesize that overall quality of life and psychological and physical functioning will be improved, potentially offering a better understanding of supportive cancer care in hematology and inferring new perspectives in psychophysiological research in cancer.

**Trial registration:**

Current randomized controlled trial was registered 29 November 2017 on Clinical Trials.gov (NCT number: NCT03356171).

## Background

### Hematologic patients

Hematologic cancers are heterogeneous diseases, defined by hematopoietic myeloid or lymphoid cell tumors, originating from the bone marrow or the lymphatic system [1]. They include numerous different diagnostics, according to the 2008 World Health Organization classifications [2] and their revised criteria in 2016 [3], from indolent and chronic lymphoid tumors to rapidly aggressive acute leukemia. The prevalence of hematologic cancers has been constantly in progress in developed countries [4]. There were approximately 230,000 new cases of hematologic cancers in Europe in 2005 and patients with these types of tumors accounted for 7% of all cancer-related deaths in that same year [5].

Hematologic patients have poorer health-related quality of life [6, 7], with substantial changes in physical, psychological, and social components due to the disease and its treatments [8]. High-dose chemotherapy is often required, and sometimes hematopoietic stem cell transplantation (HSCT) is also administered. Serious physiological complications related to treatment side-effects have been reported such as cardiomyopathy [9], neuropathy [10], and decreased immune function induced by bone marrow damage related to the chemotherapy [11]. While most HSCT recipients regain functioning to carry out activities of daily living during the year following HSCT [6, 7], patients report persistent fatigue, anxiety, and depression at their one-year follow-up which affects their physical, emotional and social functioning [7]. Three years following treatment, patients were found to have higher fatigue, dyspnea and insomnia compared to the general population [6]. These symptoms are known to negatively affect physical and cognitive functioning and general quality of life [6]. Beyond residual symptoms, persistent psychological and social problems related to self-image, distress, isolation, and fear about the future or relapse are experienced by most patients undergoing HSCT [12].

This persistent symptomatology and their long-term negative effects on overall quality of life are a major concern for these hematologic patients [6–8]. Consequently, symptom management interventions should continue after treatment cessation to support the challenge of returning to an active daily life and to their former roles in work and society [12, 13].

### Complementary therapies to enhance health-related quality of life

#### Physical exercise

Regarding hematologic patients, programs based on endurance, resistance, relaxation or stretching are well tolerated [14], even by patients before, during or after undergoing HSCT [15]. Aerobic exercise, combined or not with resistance training, improves not only physical performances such as aerobic capacity, cardiovascular fitness and muscular strength, but also fatigue and overall survival [16, 17]. Furthermore, positive effects of physical exercise on immune function, with shorter duration of neutropenia and thrombocytopenia, were measured among patients undergoing HSCT [16]. Combining aerobic and resistance training, with moderate intensity (12–14 Borg scale, 70–80% max heart rate), up to 30 min and more, 3–5 times per week over 6–12 weeks, may be the most efficient for hematologic patients after HSCT [16].

Whereas physical exercise affects physical functioning and fatigue, there is no consensus about positive effects on psycho-emotional components of quality of life [15, 18, 19]. No conclusive results have been found regarding depression, anxiety, and sleep disturbance among hematologic patients [14], and no significant results were reported about the impact of a physical exercise program on improving social functioning for patients undergoing HSCT [15]. These results suggest that physical exercise alone may be limited to improve all components of quality of life and could therefore be combined with another intervention centered on psycho-emotional functioning to optimize its efficacy in cancer rehabilitation [20].

#### Heart rate variability biofeedback

Heart rate variability biofeedback (HRVB) is a behavioral intervention in order to improve the dynamic balance of the autonomic nervous system (ANS) and to regulate emotional state [21]. It consists of a regular breathing practice at a specific frequency of approximately 6 breaths/min that produces high amplitude of heart rate variability (HRV) [22].

HRV is defined by fluctuations in the inter-beat interval between all successive heartbeats, and is characterized with R-spikes spatial intervals of the ECG (RR intervals) in non-arythmic patients. HRV reflects the dynamic interaction between the sympathetic and parasympathetic nervous systems [23–25]. It is assessed in the time domain (e.g. standard deviation of normal-to-normal R-R interval (SDNN)) and in the frequency domain (e.g. fast Fourier transform (FFT)), providing information concerning the variability of RR intervals and the underlying nervous mechanisms [26]. HRV is recognized as a biomarker for good health: a high level (in a physiological range) of HRV indicates higher resilience and an ability to face challenges, such as stressors or exercise [27]; a reduced HRV measurement is associated with cardiovascular mortality and morbidity [28] or with serious psychological disorders, such as depression [29] and anxiety [30].

Two main autonomic nervous regulatory mechanisms are associated with HRV pattern: baroreflex and respiratory sinus arrhythmia [21]. The baroreflex maintains blood pressure (BP) by increasing HR if BP decreases and vice versa [31]. Respiratory sinus arrhythmia increases HR during inspiration and decreases it during expiration [32]. These oscillatory systems can synchronize themselves at a specific resonance frequency around 0.1 Hz with respiratory rate at approximately 6 breaths/min [22]. A specific HRV pattern known as cardiac coherence or psychophysiological coherence emerges from this synchronization and is characterized by a sine wave-like HRV pattern having a peak in the low frequency region centered around 0.1 Hz on the spectral analysis and a maximal amplitude in HRV signal [21, 33, 34].

HRVB positively impacts the cardiorespiratory system by improving HRV level, gas exchange and baroreflex gain [35]. On a broader scale, HRVB may improve the balance of the ANS and the regulation of inflammatory responses [36, 37]. HRVB could also be effective to promote psychological resiliency in terms of emotional responses and recovery from environmental stressors [34, 36, 38]. This research is supported by recent studies in functional neuro-imagery that have associated HRV with some areas of the limbic system and the prefrontal cortex which are both involved in emotional state regulation [38–40]. Several clinical studies concluded that patients with various chronic diseases responded well to HRVB, having reported satisfaction and positive well-being after biofeedback practice [37, 41, 42]. Their results are promising for clinical outcomes revealing positive effects on physiological and psychological symptomatology. In addition, most of these studies determined the feasibility of HRVB and the achievement of cardiac coherence state by patients [37, 41, 42].

### Objectives and outcomes

Our protocol evaluates the superiority of an adapted physical activity (APA) program combined with HRVB (experimental group), over a program with APA only (control group). The APA program is suggested for hematologic patients as a supportive cancer care by the ACSM exercise guidelines for cancer survivors [14] and has also been recommended specifically for patients undergoing HSCT [16].

The primary objective will be to investigate the effects of a 12-week intervention of HRVB associated with an APA program versus APA only on overall quality of life measured with Quality of Life Core Questionnaire (QLQ-C30) in hematologic patients. Specifically, we will evaluate the effects on fatigue, anxiety and depression; assess clinical status with BP, progression-free survival, overall survival, and body mass index (BMI); and measure HRV level, cardiac coherence score, physical condition, satisfaction, and adherence to the protocol (to ensure the quality of support provided to patients).

### Hypothesis

Patients who participate in HRVB training in addition to an APA program versus APA only are expected to:

1. Improve their overall quality of life and reduce their overall fatigue, anxiety and depression.
2. Improve their clinical status through BP, progression-free survival, overall survival, and BMI.
3. Increase their HRV level and cardiac coherence score.

## Methods/design

### Design and setting

The APACCHE protocol is a single-centre, parallel, randomized controlled superiority trial in adult hematologic patients who have been treated for hematologic malignancy in the hematology unit of the Hospital of Reunion Island, France. Modifications of this current protocol will have to be written as a protocol amendment by the Methodology and Data Management Centre and will have to be validated by the French ethical committee.

### Study population

#### Inclusion criteria

Our study will include French-speaking patients, aged 18–70, with various forms of hematological malignancies, in post-treatment remission (full or partial) within the 6 months prior to the study, with stable hemoglobin levels (≥9 g/dl) and for whom APA is recommended by a hematologist. All patients will give their written informed consent.

#### Exclusion criteria

Patients will be excluded from the study in the following circumstances: under guardianship, medical contraindication to physical exercise, anti-arrhythmic or beta-blockers treatment and/or heart failure (left ventricular ejection fraction (LVEF) < 40% measured using echography pre or post-therapy). Patients who participate in another clinical study about non-pharmacological intervention will also be excluded to avoid potential bias in evaluation of quality of life enhancement.

### Interventions

#### APA program

**Fig. 1:**
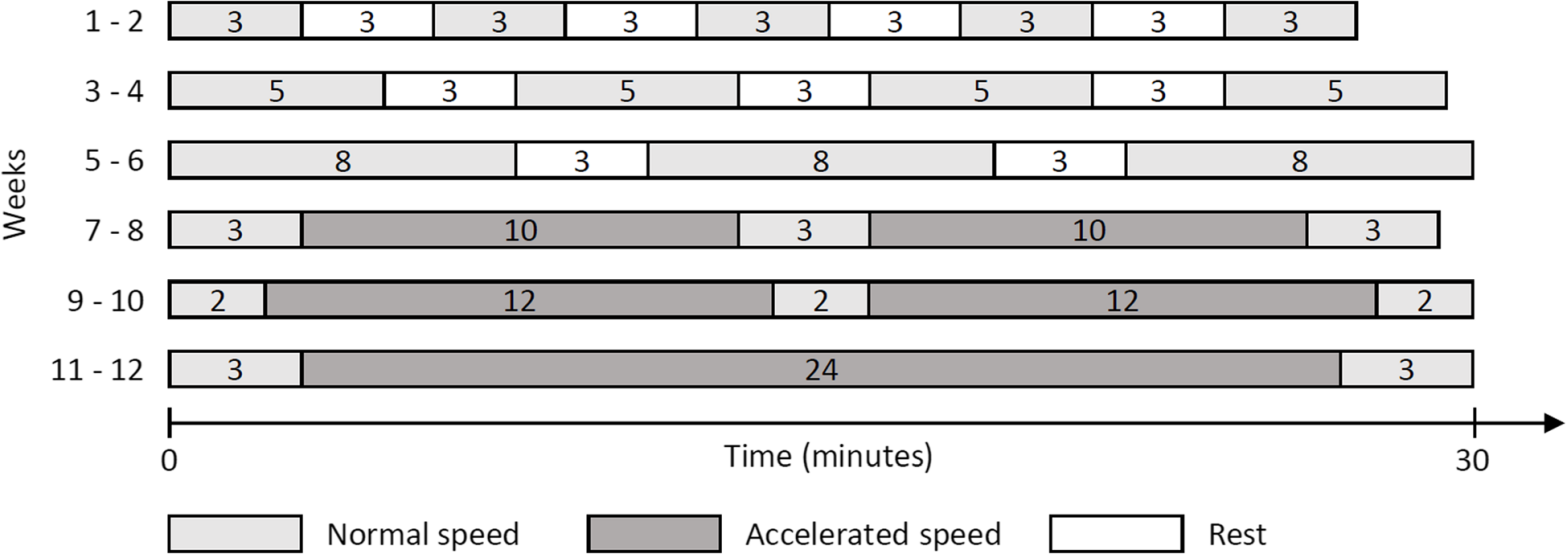
Walk protocol adapted from Mello [43]. White: rest; light grey: normal speed (7–9 Borg Scale); dark grey: accelerated speed (10–13 Borg Scale)

The APA program will consist of supervised aerobic and resistance training at moderate intensity of 12–14 on the Borg scale, corresponding to 70–80% max heart rate [16]. It will be supervised by an APA instructor during 12 weeks with 1-h sessions twice a week. The intensity will be progressive, adapted to individual capacities and functional limitations of each patient. This intervention will be done in small groups to promote exchange and conviviality and to optimize the rates of participation and compliance. Each session will be composed of four sections: 5-min warm-up, 30-min aerobic training, 20-min resistance training, and 5-min cool-down. Warm-up will include soft joint movements to prevent injuries. Aerobic training could involve a walking protocol (Fig. 1) or step aerobic exercises. Resistance training will consist of circuit training with various muscular reinforcement exercises. Finally, there will be 5-min of stretching exercises for the cool-down.

The walking protocol for weeks 1–6 will alternate between periods of walking at a comfortable speed and rest. Weeks 7–12 will include periods of walking at an accelerated speed and walking at a comfortable speed, as describe in Fig. 1. This 12-week protocol is adapted from and inspired by a treadmill program over 6 weeks previously tested in hematologic patients [44]. The walking protocol will take place on flat ground and stairs in the hospital park to promote functional rehabilitation in natural surroundings. In case of rain and to diversify the activity, step aerobics could be offered as an exercise in the fitness room of the hospital. The step aerobics will consist of choreographed movements to music. This activity requires cardiovascular adaptations and stimulates physical coordination, balance, and cognitive abilities such as memory or attention. In addition, it is a playful activity that promotes conviviality [45, 46].

Resistance training will be implemented to increase muscle strength and to reinforce stability, coordination and flexibility. Reinforcement of muscle chains, such as upper/lower limb (e.g., arm curl, squats) or lap belt (e.g., plank, modified curl-up) will be carried out with body weight, dumbbells (1–2 kg) or medicine balls (3–5 kg). These workouts will consist of a circuit training model including team challenges and games. We will closely survey all patients and pay particular attention to their form and posture to prevent pain and injuries. All of the exercises may be adapted according to each person’s abilities.

At the end of the APA program, patients will be encouraged to continue regular physical exercise at home, with friends or family, or with a club or association.

#### HRVB training

HRVB will include 10 supervised, 1-h sessions spread evenly over the course of the 12 weeks and will be supervised by the APA instructor in a dedicated room at the hospital. Sessions will consist of exercises using the Symbiofi® cardiac coherence software (SymbioCenter® technology, SymbioLine® Professional, SYMBIOFI, Loos, France) that provides various interactive workouts to control the rhythm and amplitude of respiration in order to achieve a cardiac coherence state with an elevated cardiac coherence score. To help patients achieve the cardiac coherence state, they will see their cardiac coherence score displayed on the screen along with a respiratory guide at 6 breaths/min and the feedback of HRV in real time (tachogram) from a plethysmograph pulse sensor.

The first two of the ten supervised sessions will provide exercises based on relaxation techniques to breathe consciously and focus on abdominal breathing. The following sessions will include interactive exercises with biofeedback reflecting cardiac coherence state. For example, a patient will see displayed on a screen a changing landscape from stormy to sunny weather according to the cardiac coherence score. The last HRVB sessions will include exercises that require more autonomy using only relaxing music and pictures. Each session will end with a 5-min HRV recording with respiratory guidance at 6 breaths/min to compare cardiac coherence scores from one session to the next.

At the first session, the following instruction will be given: *“You are going to participate in a 10-session program in which you will perform breathing exercises that will help alleviate your symptoms. You will have to come to the hospital center to do your training every week for 10 weeks. This intervention will be complementary to the adapted physical activity program that you will follow. I ask that you do not talk about what you do during these cardiac coherence trainings during your physical activity sessions, since some patients will not be doing this program.”*

*“We will be using a device with a pulse sensor to measure your heart rate. We will perform exercises that will allow you to control your breathing at a slower pace than usual. This breathing should produce strong, positive effects on your nervous and cardiovascular systems and help you to feel better.”*

*“The cardiac coherence training includes a part that should be done at home, with daily exercises. You should use the DODOW® device 20 minutes a day. This device is a light metronome to help you synchronize your breathing. A light is projected on the ceiling and you have to inhale when the light beam increases in brightness and to exhale when the light beam decreases in brightness. You will need to do this exercise each day at the same time, whenever you prefer. You can do this exercise just after waking up or before dinner, for instance.”*

Throughout the protocol, patients will have to practice cardiac coherence 20-min daily at home by controlling their breathing at 6 breaths/min, according to Lehrer’s protocol [47]. They will continue their home-based breathing exercises after completing the 10 supervised sessions and until the 12-week follow-up, when the study will be terminated. We will offer patients a light metronome (DODOW®, LIVLAB® technology, Paris, France) to assist in synchronizing their controlled breathing. The home-based practice will be registered in a logbook with date, duration and satisfaction, and will be monitored by the APA instructor.

### Assessments

#### Quality of life assessment

Quality of life will be evaluated with the QLQ-C30 version 3.0 developed by the European Organization for Research and Treatment of Cancer [8]. The QLQ-C30 incorporates 5 functional scales (physical, role, cognitive, emotional, and social); 3 symptom scales (fatigue, nausea/vomiting, and pain); 6 single-items (dyspnea, insomnia, appetite loss, constipation, diarrhea, and financial difficulties); and a global health subscale. Scores will be transformed to a 0–100 scale, with 100 reflecting highest functioning, highest symptomatology or highest global health, according to each scale. The minimal clinically important difference (MCID) is at least 5 points to achieve a clinically significant improvement [48].

To complement this assessment, the Medical Outcomes Study 36-Items Short-Form Health Survey (SF-36) will also be used [43]. The French validated version [49] contains 8 scales: physical functioning, role limitations (physical), bodily pain, general health, vitality, social functioning, role limitations (emotional), and mental health, which may be gathered in 2 second-order factors consisting of a mental component summary and a physical component summary. For each dimension, item scores will be coded, totaled, and transformed to a 0–100 score where the lowest score of 0 will correspond to ‘least healthy’ and the highest score of 100 will correspond to ‘healthiest’, with an estimated MCID of 3 points [50].

#### Fatigue assessment

The Multidimensional Fatigue Inventory (MFI-20) includes 20 items divided into 4 dimensions in the French version versus 5 dimensions in the English version [51]: general/physical fatigue, mental fatigue, reduced activities and motivation. Each subscale will be normalized to a 0–100 score: high score indicating a high degree of fatigue with a MCID of 3 points [52].

#### Anxiety and depression assessment

Anxiety and depression will be evaluated with the Hospital Anxiety and Depression Scale (HADS), which has been validated to measure anxiety and depression without confounding somatic symptoms of physical disorders [53]. It contains 14 items divided into 2 subscales: HADS-Anxiety and HADS-Depression of which the MCID is estimated at 1.7 points [54]. For each subscale, cutoff scores ≤7 indicates no symptoms; 8–10 signifies suspect disorder; and ≥ 11 represents significant disorder.

#### Clinical status assessment

BP, infectious events, serious infectious events, relapses, deaths, and their reasons will be collected by a hematologist as part of the standard medical surveillance. Given that a low BMI is correlated with poor survival after HSCT, BMI will be used to monitor weight fluctuations as a function of height [55]. Specifically, the BMI reference value used will be the representative average BMI value in a general population from an industrialized country (approximately 25 ± 2.5 kg/m^2^) [56].

#### HRV level and cardiac coherence score

HRV will be recorded at rest using the noninvasive SymbioLine® plethysmograph pulse sensor over a 10-min period. The recommended body posture will be sitting with knees at 90°, feet flat on the floor, hands on thighs, palms facing upward and eyes closed according to standard measurement of the short-term HRV [57]. Patients will be asked to relax and breathe normally and will have to stay seated without speaking or making any movements.

The last 5 min of HRV recording will then be analyzed with *HRVanalysis,* a free software that analyzes RR intervals (https://anslabtools.univ-st-etienne.fr) [58]. We will collect indicators of HRV to estimate the HRV level and cardiac coherence score. The SDNN indicates the level of cardiac variability, with high SDNN corresponding to high variability. The cardiac coherence score in percentage is associated with a good cardiac coherence state [26].

#### Physical condition assessment

Physical function will be evaluated with a 6-min walk test [59] and 50-ft walk test at fastest speed [60]. Muscular strength will be estimated from grip strength measured with a dynamometer for which values are correlated to general muscle mass [61]. The MCID of 6-min walk test is estimated at 25 m [62] but no MCID is known for 50-ft walk test and grip strength in chronic patients. Stability will be measured with a single limb stance test (eyes open and closed) and flexibility will be evaluated on both the lower and upper body. All of these assessments have been previously tested in hematologic patients or in the elderly; they are adapted and feasible for our study population [63–65].

The APA instructor will administrate the Global Physical Activity Questionnaire (GPAQ) to evaluate daily activities. The French version of GPAQ is validated by the World Health Organization [66] and collects information about three domains of physical activities (activity at work, travel to and from places, and recreational activity) and sedentary behavior.

#### Satisfaction and adherence to the protocol

Patient satisfaction and participation rates at APA and HRVB sessions (including information about dropout and reasons for doing so) will be monitored over the course of the study to assess the interest among hematologic patients throughout these interventions.

### Evaluation times and implementation

**Fig. 2:**
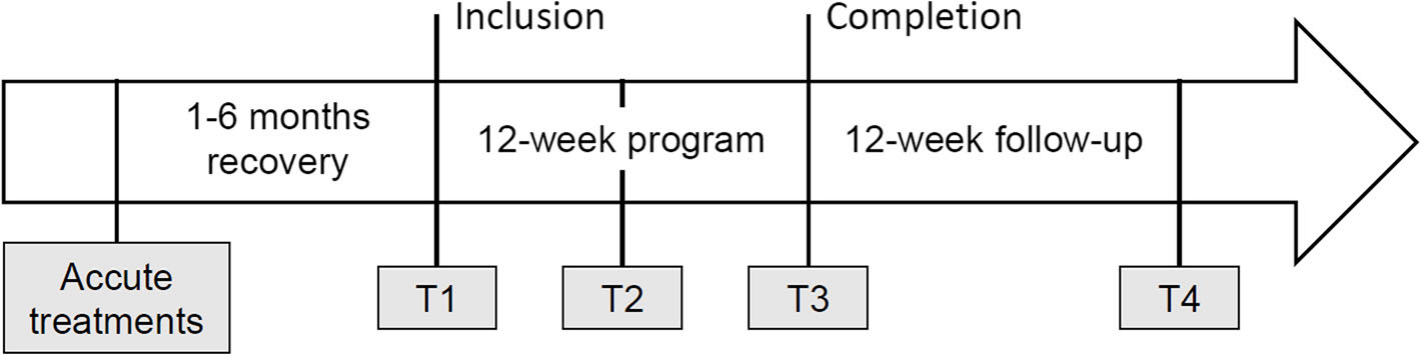
Timeline of the APACCHE study

The APACCHE protocol will include 5 evaluation sessions: an introduction about protocol and medical prescription (T0), patient screening and getting informed consent (T1), a mid-term evaluation after 6 weeks of intervention (T2), a final assessment at 12 weeks when the intervention concludes (T3), and a 12-week follow-up (T4) (Fig. 2). To ensure a high-quality and organized protocol and to coordinate the various staff members in the hematology unit, a care pathway will be implemented.

At T0, hematologists will deliver a brochure about the APA program and a briefing note regarding the protocol to potential participants. They will complete a dedicated form related to the medical prescription of the APA program including details about clinical status and specific recommendations for the APA instructor. Then, the APA instructor will contact interested patients to make a first appointment (T1).

At T1, the hematologist will obtain informed consent from the patients, and will proceed to the randomization. For study participants who will be included in the experimental group, a clear description about HRVB training will be given by the APA instructor.

At T1, T2, T3 and T4, quality of life, fatigue, anxiety and depression will be assessed by trained interviewers. Clinical status will be collected by the hematologist and HRV level, cardiac coherence score, and GPAQ will be measured by the APA instructor. Physical tests will be conducted only at T1, T2 and T3. (Table 1).

**Table 1:**
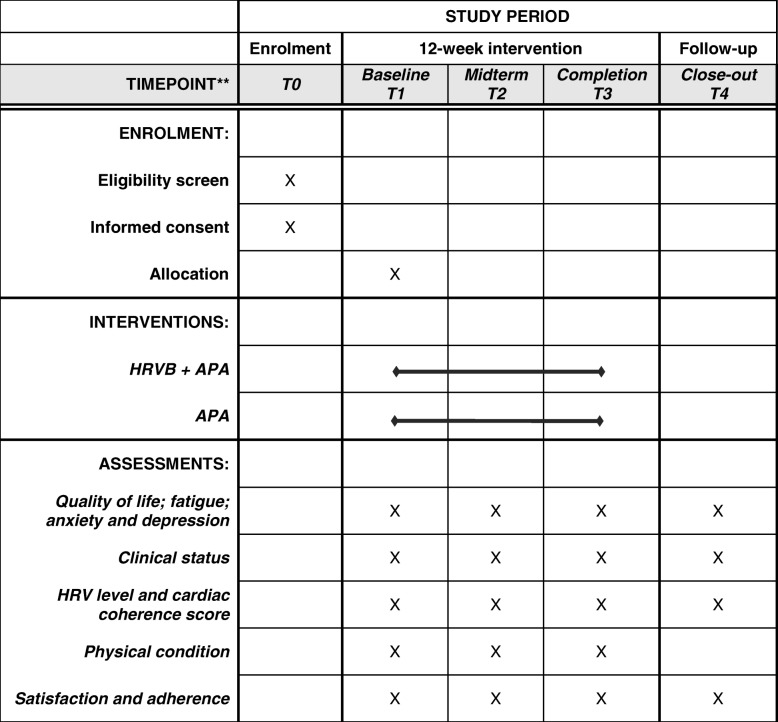
Schedule of enrolment, interventions, and assessments from SPIRIT guidelines

### Data collection and statistical analysis

#### Sample size

**Fig. 3:**
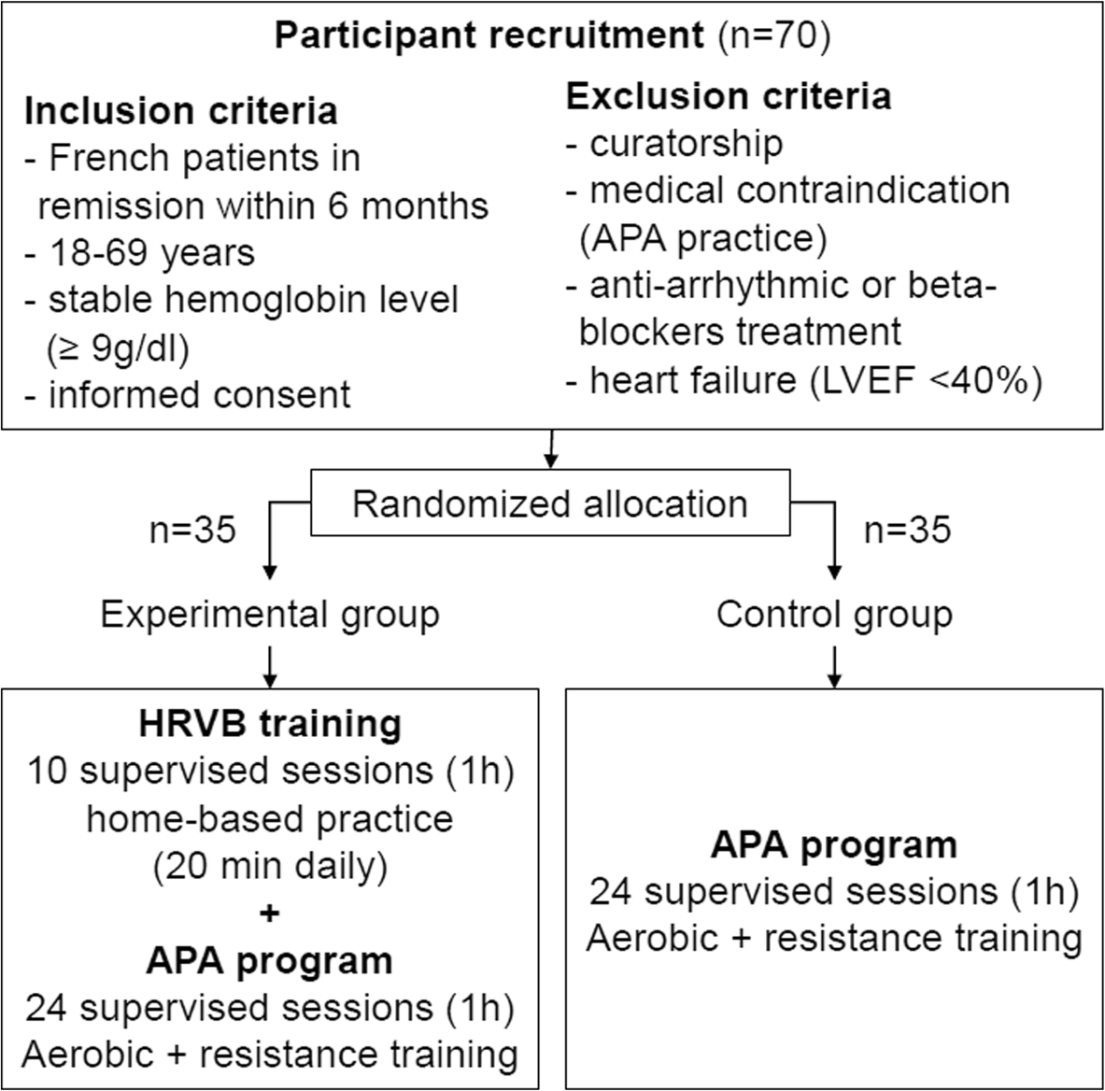
Recruitment and randomized allocation group from the CONSORT 2010 flow diagram

Based on a large study among 2800 hematologic patients [6], we expect an additional increase of 5 points, with a standard deviation of 7 points, in the QLQ-C30 score of the experimental group (HRVB+APA) after the 12-week intervention. Due to an estimated 10% missing data rate and dropout rate (abandonment, death, loss of sight), we aim to include 70 patients in the protocol (Fig. 3). They will be randomly assigned to one of two treatment groups (HRVB+APA (*n* = 35) versus APA only (n = 35)). Sample size was calculated with PASS 11® software (power analysis and sample size) to show a significant difference in improvement of quality of life in the experimental group with an alpha level of 5% and a statistical power of 80%.

#### Blinding and intervention assignment

The specificity of the HRVB and APA interventions will not allow for the use of double-blind assignments for the APA instructor and hematologists. However, we will establish a blinding on the questionnaire assessments for the trained interviewers to maintain a high level of evidence.

Randomization will be balanced in both groups, with a 1:1 allocation ratio, without stratification. The randomization list will be established by the statistician from the Methodology and Data Management Centre before the start of the research. Different sizes of randomization blocks will be used to leave the investigators blind to their size and to avoid predictable allocation. Randomized block sizes of 4,6,8 or 10 blocks will be performed on STATA®. The randomization list will then be imported into the CsRandom module of the Ennov Clinical® electronic case report form (eCRF), which the investigator will use to perform the randomization.

#### Database and data monitoring

The database will be created using EnnovClinical® software and the access will be secured (personal ID and password required) with different levels of security depending on the role of the investigator. Access will be permitted for the hematologist and the APA instructor (with limited access on their outcomes), the trained interviewers (with limited access on outcome measurement due to blinding), clinical research associate (read-only), methodologist (read-only), and biostatistician (read-only). The clinical research associate of the Methodology and Data Management Centre will visit the hematology unit twice during the protocol and once at the end to verify all consent forms, compliance with established protocol and procedures, and data quality in the eCRF. A data validation plan, jointly defined between the coordinating investigator and the Methodology and Data Management Centre, will be developed and will describe in detail the controls to be performed for each variable. Once the data has been entered, data consistency will be evaluated, and any omissions or inconsistencies will be declared.

Adverse effects and serious adverse effects will be identified according to standard guidelines, recorded in the eCRF and transmitted to the research partners by the main investigator. Patient safety is paramount and any complications and adverse events will be reported and analyzed.

#### Statistical analysis

To evaluate effectiveness of HRVB, we will compare the average evolutions (T3-T1 difference score) between the experimental group (HRVB+APA) and the control group (APA). Student’s t-test will be performed for the different dimensions and scale sub-scores of each questionnaire, as well as cardiac coherence score and SDNN. An analysis of longitudinal data collected at T1, T2, T3 and T4 will be conducted using a linear mixed model with structured covariance residuals. The bivariate survival analysis (death and / or relapse, survival without serious infectious events) will be carried out using the Kaplan-Meier method. Group comparison will be calculated using the log-rank test.

All hypotheses will be tested in a single analysis, intention to treat (according to the group assigned by the randomization), bilaterally at the threshold α = 5%. The analyses will be performed with SAS® (version 9.4, SAS Institute Inc., Cary, NC, USA). At the end of the research study, investigators will anonymize the collected data and results will be submitted to the research partners. The statistician, methodologist and investigators will have access to the final dataset. An article in a journal indexed on Medline will be carried out in post-trial.

## Discussion

Previous studies have investigated the effectiveness of HRVB compared to physical exercise [67–69]. To the best of our knowledge, there have been no studies about the added value of HRVB on physical exercise to enhance overall quality of life. While recent studies about HRVB training have demonstrated the feasibility and the effectiveness of HRVB in chronic patients [37, 41, 42], it has not yet been done in cancer patients, namely in hematology. Most clinical studies conclude that HRVB can reduce symptomatology of chronic disease and improve HRV level, supporting our hypothesis that HRVB may be feasible and effective in recovery of hematologic patients.

We hypothesize that HRVB associated with an APA program will be more effective than an APA-only program in improving quality of life during post-treatment in adult hematologic patients and expect the following outcomes from this experimental group: i) significant improvements in overall quality of life and a reported decrease in overall fatigue, anxiety and depression; ii) increased HRV levels, reflecting a better physiological regulation through autonomic control of the heart; iii) better BMI and BP measurements; and iv) less infectious events, relapses and deaths. All of these variables should be improved between T1 and T3. The results recorded at T3 could be sustained through T4, especially if patients are persistent with their home-based practice for both cardiac coherence and physical exercise.

All participating patients will be in remission for hematologic malignancies that have required hospitalization, caused sudden disruptions to their day-to-day lives, and/or have required particular aggressive treatments with high-dose chemotherapy, affecting patients’ overall quality of life. To avoid age-related effects, we have chosen a within-subject design and will exclude patients over 70 years.

Our short-term HRV recording protocol is based on the recommendations from Task Force [70] and more recently from Laborde [57], who has suggested guidelines for psychophysiological research. Although electrocardiogram is more accurate, we use the pulse sensor to assess pulse-to-pulse interval data which accurately approximates the inter-beat interval [71]. To analyze HRV data in time-domain and frequency domain, *HRVanalysis* will be used [58].

We place particular importance on describing the intervention and detailing how it will proceed to identify modalities of an effective program for assisting with cancer recovery, especially after hematologic treatments. This proceeding should enhance the implementation of a care pathway in the hematology unit at the Hospital of Reunion Island and facilitate its reproduction in other health care facilities.

In conclusion, the results of the APACCHE study should demonstrate effects of HRVB training associated with an APA program on physical functioning and emotional state in treated hematologic patients. We anticipate that overall quality of life in both psychological and physical capacities will improve. Results of this protocol will be published in the hope that our research outcomes may produce relevant knowledge about supportive cancer care in hematology and may infer new perspectives in psychophysiological research in cancer.

## Data Availability

After the completion of the current protocol, data and materials will be available from the corresponding author upon reasonable request.

## Abbreviations

APACCHE: Adapted Physical Activity and Cardiac Coherence in Hematologic patients
APA: Adapted Physical Activity
HRVB: Heart rate variability biofeedback
ANS: Autonomic nervous system
HRV: Heart rate variability
HSCT: Hematopoietic stem cell transplantation
SDNN: Standard deviation of normal-to-normal R-R interval
FFT: Fast Fourier transform
BP: Blood pressure
QLQ-C30: Quality of Life Core Questionnaire
BMI: Body mass index
LVEF: Left ventricular ejection fraction
MCID: Minimal clinically important difference
SF-36: Short-Form Health Survey
MFI-20: Multidimensional Fatigue Inventory
HADS: Hospital Anxiety and Depression Scale
GPAQ: Global physical activity questionnaire
eCRF: electronic case report form
